# A systematic literature review of the clinical signs and symptoms of veno‐occlusive disease/sinusoidal obstruction syndrome after haematopoietic cell transplantation in adults and children

**DOI:** 10.1002/jha2.612

**Published:** 2022-11-29

**Authors:** James Angus, Shannon Connolly, William Letton, Chris Martin, Alison Martin

**Affiliations:** ^1^ Jazz Pharmaceuticals Oxford UK; ^2^ Crystallise Ltd. Essex UK

**Keywords:** adult, children, diagnosis, sinusoidal obstruction syndrome, veno‐occlusive disease

## Abstract

Veno‐occlusive disease/sinusoidal obstruction syndrome (VOD/SOS) is a rare, serious complication following haematopoietic cell transplantation (HCT). This systematic literature review evaluated differences in clinical manifestations of VOD/SOS post‐HCT in adults and children. Medline and Embase were searched up to 4 March 2021 for reports of VOD/SOS post‐HCT; VOD/SOS diagnostic guidelines were included. Publications were evaluated based on inclusion of five cardinal clinical features of VOD/SOS (ascites, hepatomegaly, hyperbilirubinaemia, right upper quadrant [RUQ] pain and weight gain ≥5%). Overall, 204 publications were included. At diagnosis, hyperbilirubinaemia was more common in adults (93%) versus children (82%), weight gain ≥5% and hepatomegaly were more common in children (86%, 89%) versus adults (73%, 76%) and ascites and RUQ pain were similar between age groups. While 40% of cases had all five cardinal features, age was not a substantial determinant of the likelihood of missing any single specific feature. The proportion of cases, where hyperbilirubinaemia was the first recorded feature, was higher in children versus adults; weight gain and RUQ pain appeared first in a greater proportion of adults versus children. VOD/SOS diagnosis can be challenging; features may not present in a distinct sequence. This necessitates continuous vigilance by those involved in patient monitoring post‐HCT.

## 1 INTRODUCTION

Veno‐occlusive disease/sinusoidal obstruction syndrome (VOD/SOS) is a potentially life‐threatening complication of haematopoietic cell transplantation (HCT) conditioning that can also develop after high‐dose chemotherapy. It is associated with mortality rates of >80% in severe disease with supportive care alone [1, 2]. The incidence of VOD/SOS post‐HCT generally ranges from ∼5% to 14%, and potentially higher depending on patient characteristics, transplant setting and diagnostic criteria used [1]. The incidence of VOD/SOS is 20% overall in children and can be up to 60% in high‐risk patients, such as those with osteopetrosis, high‐risk neuroblastoma, thalassaemia and congenital macrophage activation syndrome [3]. Early diagnosis in both adults and children is crucial, as prompt appropriate treatment can improve prognosis [4].

The clinical signs and symptoms of VOD/SOS, which were first characterised more than three decades ago, include ascites, hepatomegaly, hyperbilirubinaemia, rapid weight gain and right upper quadrant (RUQ) pain [5, 6]. Several sets of diagnostic criteria have been subsequently developed that, over time, have evolved in regard to the requirements and/or thresholds for these signs and symptoms to be present to warrant a diagnosis of VOD/SOS. Current diagnostic criteria require different combinations and definitions of these signs and symptoms for diagnosis and severity grading of VOD/SOS [1], and treatment guidelines from various organisations differ in their recommendations for using the diagnostic criteria [3, 7, 8]. Differential diagnosis of VOD/SOS can be difficult because signs and symptoms overlap with other post‐transplant complications; accurate and timely diagnosis is further complicated by the many patient‐ and transplant‐related VOD/SOS risk factors, the sometimes insidious onset of disease and the dynamic presentation of signs and symptoms, some of which may differ by patient age [1].

We undertook this systematic literature review to evaluate differences in the reported clinical manifestations of VOD/SOS after HCT in adults and children.

## 2 METHODS

MEDLINE and Embase were searched up to 4 March 2021 for studies of VOD/SOS post‐HCT and were supplemented with guidelines on VOD/SOS diagnosis and management. The detailed search strategy is reported in the **Appendix Tables** **1** **through** **4**. English language reports of observational and database studies were included if they studied adults and/or children with any HCT‐related disease, therapies aimed at preventing or treating VOD/SOS or HCT‐associated VOD/SOS outcomes. All abstracts were screened independently by two researchers using the inclusion criteria shown in the Appendix. Studies with <5 patients who had VOD/SOS were indexed separately as case studies, while studies that reported on VOD/SOS occurrence in a clinical trial, where the diagnosis would have been based on a protocol rather than real‐world practice, were not included. Publications were shortlisted for inclusion in the main systematic review, using the following criteria: (1) studies reporting change in a diagnostic factor over time; (2) studies reporting the proportion of patients with features relating to the diagnostic criteria; (3) studies reporting the proportion of patients with other biomarkers or features not included in the diagnostic criteria; (4) guidelines giving recommendations on diagnosis; (5) studies that assessed how many patients have features that match the diagnostic criteria; and (6) studies that compared features of VOD/SOS with a differential diagnosis (e.g., graft‐vs.‐host disease). Publications were evaluated based on inclusion of features related to VOD/SOS diagnostic criteria, including the five cardinal features (i.e., ascites, hepatomegaly, hyperbilirubinaemia [bilirubin ≥2 mg/dl or ≥34.2 μmol/L], weight gain ≥5% and RUQ pain) and changes in diagnostic features over time.

### 2.1 Data analysis

In cohort studies of multiple cases, an unweighted mean was calculated for the proportion of patients with each VOD/SOS feature; for individual case studies, the number of case reports in which the feature was present was divided by the total number of cases in which any feature was reported.

#### 2.1.1 Data sharing

All relevant data are provided within the manuscript and supplemental files. Jazz Pharmaceuticals has established a process to review requests from qualified external researchers for data from Jazz‐sponsored clinical trials in a responsible manner that includes protecting patient privacy, assurance of data security and integrity, and furthering scientific and medical innovation. Additional details on Jazz Pharmaceuticals data sharing criteria and process for requesting access can be found at: https://www.jazzpharma.com/science/clinical‐trial‐data‐sharing/. All authors had access to the clinical trial data relevant for the publication.

## 3 RESULTS

### 3.1 Literature search results

**FIGURE 1:**
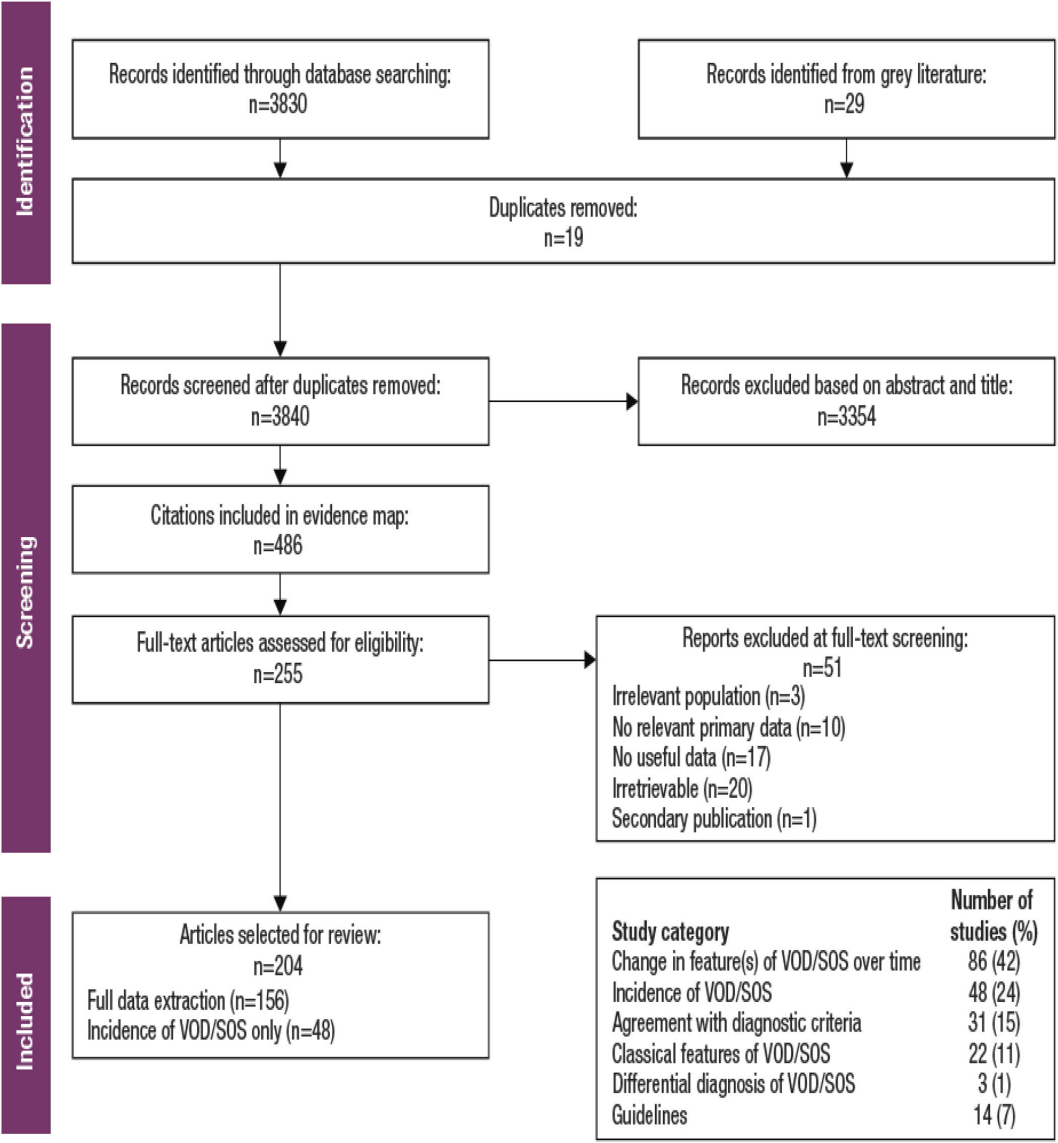
Systematic literature review article identification and selection. VOD/SOS, veno‐occlusive disease/sinusoidal obstruction syndrome

The total number of abstracts screened after de‐duplication was 3840; 204 publications were included in the systematic literature review (**Figure** **1**). A subsequent additional search for treatment guidelines yielded seven sets of diagnostic criteria and 21 guideline publications (**Appendix** **Tables** **5** and **6**).

### 3.2 Literature review results

#### 3.2.1 Presentation of VOD/SOS cardinal features

The findings for the five cardinal features of VOD/SOS are summarised in **Table** **1**. In the overall analysis population (all ages) in cohort and case studies, respectively, hyperbilirubinaemia was present in 82% and 79%, ascites in 61% and 59%, hepatomegaly in 76% and 47%, weight gain in 87% (any, in cohort studies) and 63% (≥5%, in case studies) and RUQ pain in 61% and 21% of patients. The proportion of cases for which each feature was reported as the first manifestation overall was 39% for hyperbilirubinaemia, 31% for ascites, 44% for hepatomegaly, 40% for weight gain and 50% for RUQ pain.

**TABLE 1:** Summary of overall findings for the five cardinal features of VOD/SOS

**Sign/Symptom**	**Children**	**Adults**	**Overall**	
			**Cohort**	**Case**
**Ascites**				
Proportion present	78%	77%	61%	59%
Proportion of occurrences reported as first sign	40%	6%	31%	
**Hepatomegaly**				
Proportion present	89%	76%	76%	47%
Proportion of occurrences reported as first sign	45%	93%	44%	
**Hyperbilirubinaemia**				
Proportion present	82%	93%	82%	79%
Proportion of occurrences reported as first sign	31%	14%	39%	
**RUQ pain**				
Proportion present	82%	79%	61%	21%
Proportion of occurrences reported as first sign	50%	85%	50%	
**Weight gain ≥2%**				
Proportion present	93%	91%	‒	–
**Weight gain ≥5%**				
Proportion present	86%	73%	‒	63%
**Weight gain, any**				
Proportion present	‒	‒	87%	‒
Proportion of occurrences reported as first sign	22%	54%	40%	

Abbreviations: RUQ, right upper quadrant; VOD/SOS, veno‐occlusive disease/sinusoidal obstruction syndrome.

**FIGURE 2:**
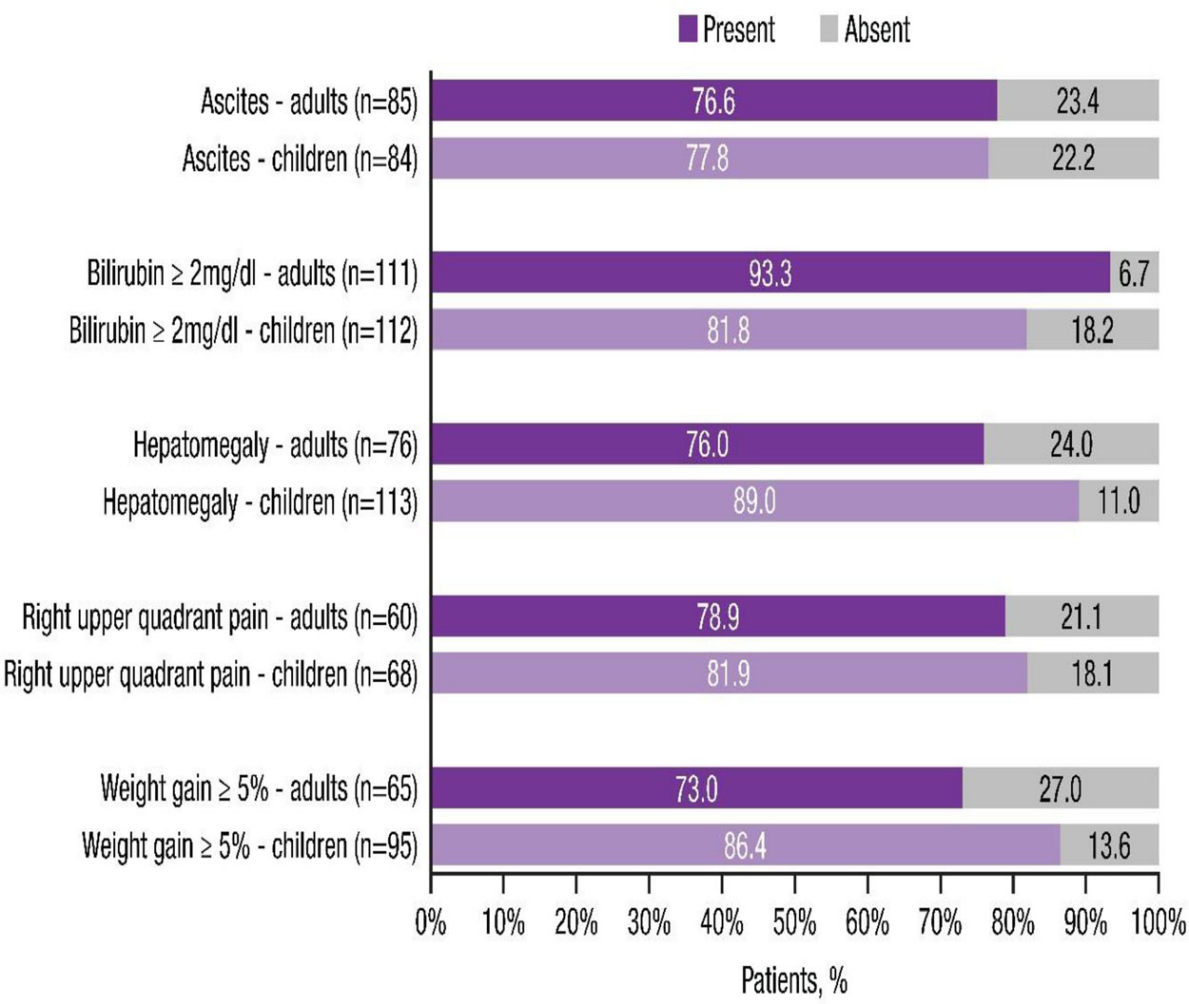
Presence or absence of five key cardinal features of VOD/SOS at diagnosis in adults and children. VOD/SOS, veno‐occlusive disease/sinusoidal obstruction syndrome

The presence or absence of each of the five cardinal features of VOD/SOS in adults and children is shown in **Figure** **2**. Hyperbilirubinaemia was more common in adults (93%) compared with children (82%). Furthermore, although VOD/SOS without elevated bilirubin does occur in adults, patients lacking hyperbilirubinaemia were generally younger compared with patients lacking ascites (children, 22%; adults, 23%) or hepatomegaly (children, 11%; adult, 24%). Weight gain ≥5% and hepatomegaly were both seen in greater proportions of children (86% and 89%, respectively) compared with adults (73% and 76%, respectively). There were no notable differences between adults and children in the occurrence of ascites or RUQ pain. In terms of feature absence by age, while only 40% of cases overall had all five cardinal features of VOD/SOS, age was not a substantial determinant of the likelihood to be missing any single specific feature.

**FIGURE 3:**
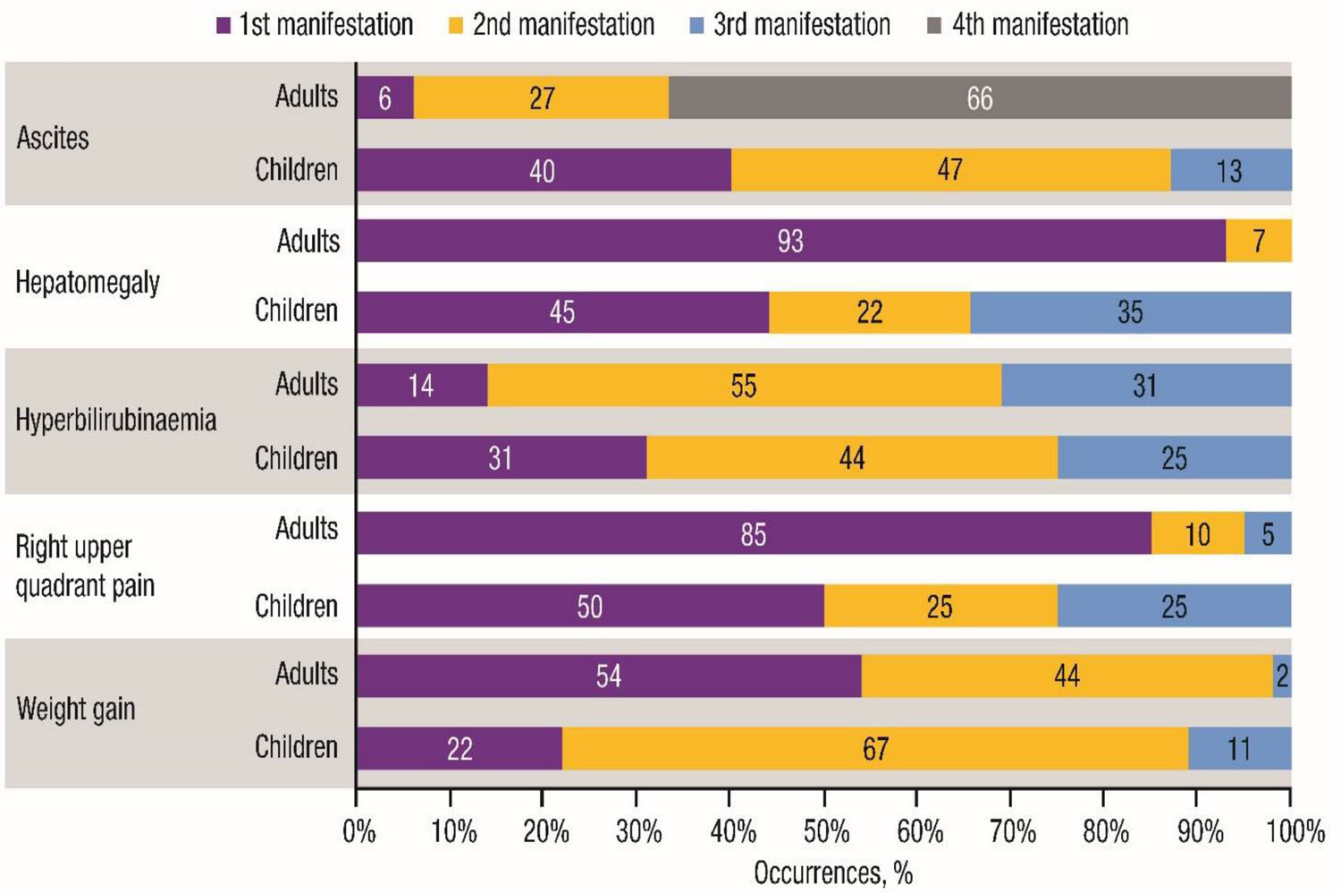
Order of appearance of the five key features of VOD/SOS. VOD/SOS, veno‐occlusive disease/sinusoidal obstruction syndrome. Figure shows the order of appearance of each feature when the feature was present. For each report, ≥1 feature could count as a first/subsequent manifestation (e.g., if a report listed ascites and hepatomegaly as the first manifestations, both were counted as first). To calculate percentages, the denominator was the total number of occurrences of each feature.

The order of appearance of the five key features of VOD/SOS in adults and children is shown in **Figure** **3**. The proportion of cases where hyperbilirubinaemia was the first recorded feature of VOD/SOS was 31% in children and 14% in adults. When weight gain was present, it appeared as the first manifestation in 54% of adults versus 22% of children; it was more likely to appear as the second manifestation in children, suggesting that VOD/SOS‐related weight gain may be less easily detected or develops later in children. Comparison of peak reported weight gain values showed a higher peak gain in children versus adults (mean 12.8% gain vs. 8.7% gain, respectively). RUQ pain appeared first in 85% of adults compared to 50% of children, suggesting that it presents later in children in relation to other symptoms or is more difficult to detect. There was no notable difference between adults and children on the day post‐HCT when any of the VOD/SOS features were first detected; the median differences of all features did not exceed 4 days.

**FIGURE 4:**
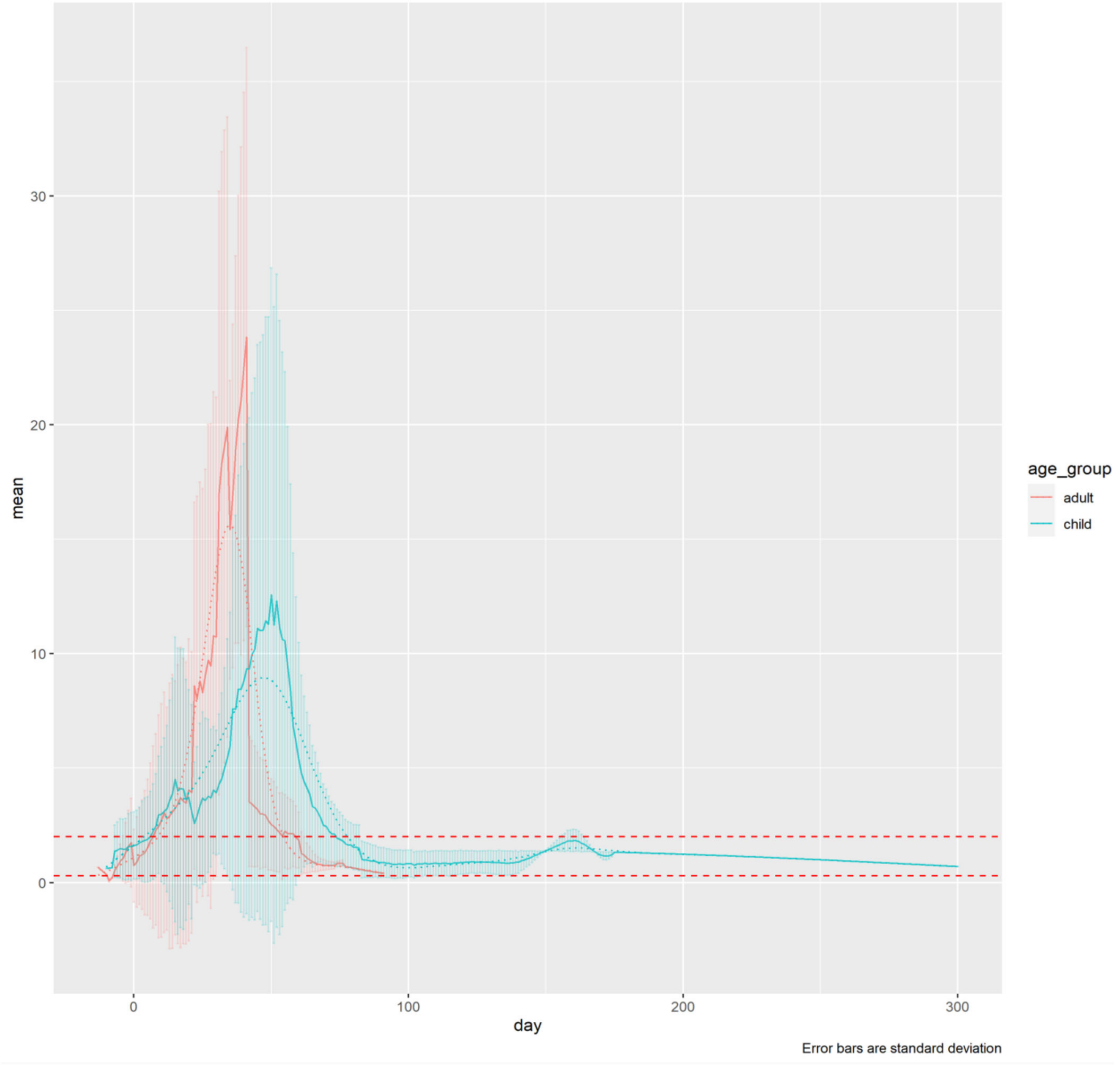
Averaged bilirubin timeseries data for cases/cohorts split into child and adult age groups. HCT, haematopoietic cell transplantation. Timeseries data were smoothed and interpolated, and then a mean value was estimated at each time point, weighted by the number of patients. Error bars represent standard deviation. The x axis ‘Day’ refers to days since HCT. The y axis ‘Value’ refers to measured bilirubin in mg/dl. Dashed red lines show the estimated healthy range.

Thirty‐six case studies reported bilirubin levels over time. Overall, the earliest abnormal values were reported from 2 days before HCT, with most patients showing a steep increase in bilirubin levels >2 mg/dl (>34.2 μmol/L) from Day 2 to Day 26 after HCT. In 11 cohort studies, mean bilirubin levels rose to >2 mg/dl (>34.2 μmol/L) by Day 8 in all but 1 group. The averaged timeseries data for bilirubin levels appeared to peak earlier and to have a higher peak for adults versus children (**Figure** **4**).

#### 3.2.2 Presentation of other abnormalities associated with VOD/SOS

Some studies also reported on the appearance of abnormalities other than the five cardinal features of VOD/SOS, the most common being abnormal levels of aspartate transaminase (AST)/serum glutamic oxaloacetic transaminase (SGOT; reported in 19 cases), abnormal levels of alanine transaminase (ALT)/serum glutamic pyruvic transaminase (SGPT; 17 cases) and abnormal levels of protein C (14 cases). Abnormal AST/SGOT, ALT/SGPT and protein C were reported as the first manifestation of VOD/SOS in 42%, 59% and 79% of their respective occurrences. From the 16 case‐study publications in which they were reported, AST levels were most commonly raised from Day 1 after HCT, while ALT values were typically raised from Day 7 after HCT. There were insufficient data to compare the occurrence of these abnormalities between children and adult patients. Abnormal platelet levels and thrombocytopaenia were reported in six cases and five cases, respectively. Overall, thrombocytopaenia was reported in 70% of patients in cohort studies and 9% of patients in case studies. Abnormal platelet levels and thrombocytopaenia were reported as the first manifestation in two cases each. Across case and cohort studies, platelet counts were usually below the normal range from 8 days before HCT, and patients who developed VOD/SOS often had refractory thrombocytopaenia, which persisted up to Day 106 after HCT. Across cohort studies, reduced platelet count or thrombocytopaenia was reported in 68% of children (three studies) and 48% of adult patients (two studies).

### 3.3 Guideline review results

Seven sets of diagnostic criteria and 21 guideline publications were identified (**Appendix** **Tables 5 and** **6**). Considerable variation was observed between diagnostic criteria in the combination and thresholds of signs and symptoms required for VOD/SOS diagnosis (**Appendix** **Table** **5**). For example, the paediatric European Society for Blood and Marrow Transplantation (EBMT) criteria require the presence of ≥2 of the following: unexplained consumptive and transfusion‐refractory thrombocytopaenia; otherwise unexplained weight gain on 3 consecutive days despite the use of diuretics or a weight gain 45% above baseline value; hepatomegaly (best if confirmed by imaging) above baseline value; ascites (best if confirmed by imaging) above baseline value; rising bilirubin from a baseline value on 3 consecutive days; or bilirubin ≥2 mg/dl within 72 h. The modified Seattle criteria require any two of the cardinal signs of hyperbilirubinaemia, hepatomegaly/RUQ pain or weight gain. The paediatric EBMT criteria specify rising bilirubin from a baseline value on 3 consecutive days or bilirubin ≥2 mg/dl (≥34.2 μmol/L) within 72 h, while the modified Seattle criteria require only bilirubin >2 mg/dl (>34.2 μmol/L). Some diagnostic criteria require a mandatory elevated bilirubin for VOD/SOS that develops within 21 days after HCT (e.g., Baltimore and EBMT), resulting in the possibility of not diagnosing VOD/SOS cases without elevated bilirubin.

Some diagnostic criteria have no time limit for onset of signs and symptoms (e.g., adult/paediatric EBMT and Cairo), whereas others are limited to within 20 or 21 days (e.g., Baltimore, modified Seattle and hybrid Baltimore/Seattle). Analysis of 46 complete adult cases from the data extraction showed that the proportion of cases that were identified using the different criteria ranged from 43% to 57% for the time‐limited criteria and 78% to 96% for the non‐time‐limited criteria. However, when diagnosing VOD/SOS within 21 days, the EBMT criteria failed to diagnose seven cases, and the Cairo criteria failed to diagnose two cases, which were diagnosed using ≥1 of the time‐limited criteria. Analysis of 68 complete paediatric cases from the data extraction showed that the proportions of cases that were identified using the different criteria were 50% for the time‐limited criteria (modified Seattle) and 51% for the non‐time‐limited criteria (paediatric EBMT). However, the non‐time‐limited criteria failed to diagnose 11 cases that were diagnosed using the time‐limited criteria. Overall, non‐time‐limited criteria detected more late‐onset cases compared to time‐limited criteria, while time‐limited criteria may detect more cases within the set timeframe.

The Baltimore and the modified Seattle criteria are dominant in the treatment guidelines, recommended in 12 and 10 of the guideline publications, respectively (**Appendix** **Table** **6**). The EBMT criteria are recommended in six guideline publications, while the paediatric EBMT criteria are recommended in four guidelines.

Guideline recommendations on monitoring for VOD/SOS features were limited and variable; for example, monitoring of serum bilirubin is recommended in five guidelines, weight in nine guidelines, ascites in five guidelines, hepatomegaly in four guidelines, abdominal pain in three guidelines and platelet levels in three guidelines.

## 4 DISCUSSION

VOD/SOS is a potentially life‐threatening complication of HCT that requires early diagnosis in both adults and children. This systematic literature review evaluated differences in the reported clinical manifestations of VOD/SOS after HCT in adults and children. Results demonstrated that the presentation of VOD/SOS symptoms post‐HCT varied among patients overall and specifically between adults and children. While hyperbilirubinaemia was more common in adults than in children, it was frequently the first recorded feature of VOD/SOS in children. The timeseries analysis shows that, on average, hyperbilirubinaemia may occur later in children than adults following HCT and may have lower average peak values in children. This suggests that anicteric VOD/SOS may be more common in children and that hyperbilirubinaemia may be delayed and less severe in children than in adults. In children, weight gain was most likely to appear as the second manifestation. Although a greater proportion of children show weight gain with VOD/SOS than adults, VOD/SOS‐related weight gain either develops later in children or is less easily detected early in the disease course in children than in adults. Ascites was the most commonly missing key feature in both adults and children. RUQ pain appeared first in most adults but was more likely to be the second or third feature to manifest in children, suggesting that RUQ pain develops later in children, or that it is identified less quickly than in adults.

Overall, the proportion of patients diagnosed with VOD/SOS who experienced each feature varied considerably, with no single feature reported in >87% of patients with VOD/SOS. The absence of a key feature, therefore, does not mean that the patient does not have VOD/SOS. Key features of VOD/SOS appeared first in no consistent pattern, with each feature being the first event detected in approximately 30% to 50% of patients. Differential diagnosis, for example distinguishing VOD/SOS from graft‐versus‐host disease, remains a challenge [7]. Our results showed that some abnormalities associated with VOD/SOS can first manifest from a few days before HCT to >30 days after HCT. The trajectory of change can be steep once an initial abnormality has developed; in particular, rapid rises in bilirubin levels have been previously associated with increased mortality risk and indication of severe VOD/SOS. Our findings suggest that frequent monitoring for VOD/SOS is advisable to facilitate early diagnosis and initiation of treatment.

No single set of guidelines diagnosed every case of VOD/SOS in either adult or paediatric cases. While all criteria use some combination of hyperbilirubinaemia, weight gain, hepatomegaly, RUQ pain and ascites to diagnose VOD/SOS, they differ in being either time‐limited (within 20–21 days) or non‐time‐limited as well as in the inclusion of additional signs and symptoms. In particular, the use of time‐limited criteria may limit the detection of late‐onset VOD/SOS. We cannot draw conclusions about the specificity of any set of diagnostic criteria as it is not possible to determine how many false positives they might generate when considering non‐VOD/SOS cases.

Review of VOD/SOS treatment guidelines indicated that the Baltimore and the modified Seattle criteria were most often recommended for diagnosis. Newer diagnostic criteria have attempted to address potential shortcomings in these criteria. The EBMT criteria addressed the difficulty in achieving early diagnosis and severity assessment of VOD/SOS with the previous criteria by introducing a severity grading scale that incorporated rate of change in bilirubin levels, liver and renal function and weight; these criteria also enabled the detection of late‐onset VOD/SOS by removing the requirement for signs and symptoms to be present 20 to 21 days after HCT [9]. The paediatric EBMT criteria acknowledged the differences in clinical presentation of the disease between children and adults by the addition of unexplained consumptive and transfusion‐refractory thrombocytopaenia as a diagnostic sign and of daily measurements of weight, rather than a single measurement [10]. The criteria of Cairo et al. incorporate more signs and symptoms as well as advances in diagnostic procedures, such as liver ultrasonography and Doppler, to assess ascites and portal venous flow [11].

The findings of our review are associated with certain limitations. Older studies included in our review report outcomes following VOD/SOS treatment regimens that may not reflect current clinical practice. In addition, because of inconsistencies in reporting, we were not able to differentiate the features of mild VOD/SOS from those of more severe disease. While changes in features of VOD/SOS over time were often better described in case reports than in cohort studies, published case reports may be likely to describe unusual cases considered noteworthy, and so less likely to represent the more typical patient. Data for patients who had an HCT but did not develop VOD/SOS are not available for comparison; a registry that tracks specific conditions, such as VOD/SOS and its signs and symptoms in patients who have an HCT, would be beneficial to understand trends in the appearance of VOD/SOS features. Abnormalities outside the five cardinal features of VOD/SOS were, in many cases, the focus of the research and, thus, often assessed in only a small number of publications in which researchers actively sought that abnormality; data on these additional features are therefore more difficult to incorporate into guidance on the early detection of VOD/SOS.

In conclusion, this systematic literature review demonstrated that presentation of VOD/SOS symptoms after HCT varied among patients overall, and specifically between adults and children. Notably, while anicteric VOD/SOS was reported in adults, it appeared to be more common at diagnosis in children. VOD/SOS features were not reported in a distinct sequence, requiring continuous vigilance and a high index of suspicion for VOD/SOS by those involved in patient monitoring post‐HCT.

## AUTHOR CONTRIBUTIONS

James Angus designed the study, contributed to writing and editing the manuscript and gave final approval to submit. Shannon Connolly conducted the analysis of atypical VOD, including differences between adults and children. William Letton performed the signs and symptoms presence and timeseries analyses. Chris Martin was responsible for the design and management of the analysis, conducted the guidelines review and contributed to the writing of the manuscript. Alison Martin designed, contributed to and oversaw all phases of the literature review and also contributed to editing the manuscript.

## CONFLICT OF INTEREST

James Angus is an employee of and holds stock ownership and/or stock options in Jazz Pharmaceuticals. Shannon Connolly, William Letton, Chris Martin and Alison Martin are or were employees of Crystallise Ltd., which received funding from Jazz Pharmaceuticals to conduct this literature review.

## Supporting information

Supporting Information

## Data Availability

All relevant data are provided within the manuscript and supplemental files. Jazz has established a process to review requests from qualified external researchers for data from Jazz‐sponsored clinical trials in a responsible manner that includes protecting patient privacy, assurance of data security and integrity, and furthering scientific and medical innovation. Additional details on Jazz Pharmaceuticals data sharing criteria and process for requesting access can be found at: www.jazzpharma.com/science/clinical‐trial‐data‐sharing/. All authors had access to the clinical trial data relevant for the publication.
