# Supplementary material for: A systematic literature review of the clinical signs and symptoms of veno‐occlusive disease/sinusoidal obstruction syndrome after haematopoietic cell transplantation in adults and children
Source: EJHaem. 2022 Nov 29;4(1):199–206. doi: 10.1002/jha2.612 (PMC9928780; doi:10.1002/jha2.612)
Supplement: Supplementary file 1 — Supporting Information [file JHA2-4-199-s001.docx]

**Appendix**

***Search strategy***

**Appendix Table 1. MEDLINE and Embase search strategy.^a^**

| **Number** | **Search string** | **Hits** |
| --- | --- | --- |
| 1 | 'liver vein obstruction'/exp OR 'liver vein obstruction' OR 'liver venoocclusive disease'/exp OR 'liver venoocclusive disease' OR 'veno-occlusive disease' OR 'venoocclusive disease' OR 'sinusoidal obstruction' OR ‘hepatic veno-occlusive disease’ | 29,324 |
| 2 | 'stem cell transplantation' OR 'h?ematopoietic stem cell' OR sct:ab,ti OR hsct:ab,ti OR conditioning:ab,ti OR myeloablati*:ab,ti OR 'bone marrow transplantation'/exp OR 'bone marrow transplantation' OR bmt:ab,ti | 328,662 |
| 3 | 1 AND 2 | 4617 |
| 4 | 3 limited to studies in humans, with abstracts, published in English | 3830 |

^a^Conducted on 4 March 2021.

**Appendix Table 2. Grey literature search strategy.**

| **Guideline** | **Website link** | **Date** | **Hits** |
| --- | --- | --- | --- |
| European Society of Medical Oncology (ESMO) | www.esmo.org/ | 03/03/2021 | 15 |
| European Society for Blood and Marrow Transplantation (EBMT) | www.ebmt.org | 03/03/2021 | 3 |
| National Comprehensive Cancer Network (NCCN) | www.nccn.org/ | 03/03/2021 | 11 |
| Gruppo Italiano per il Trapainto di midollo Osseo, cellule staminale emopoietiche e terapia cellulare (GITMO) | www.gitmo.it/ | 03/03/2021 | 0 |
| Pediatric Acute Lung Injury and Sepsis Investigators Network (PALISI) | www.palisi.org/ | 03/03/2021 | 0 |
| British Society of Blood and Marrow Transplantation and Cellular Therapy (BSBMTCT) | www.bsbmtct.org | 03/03/2021 | 0 |

Grey literature generally refers to materials and research produced by organizations outside of the traditional commercial or academic publishing and distribution channels. All guidelines were searched for management of relevant diseases.

**Appendix Table 3. Inclusion and exclusion criteria.**

| **Criterion** | **Inclusion criteria** | **Exclusion criteria** |
| --- | --- | --- |
| Population | Adults or children with any disease for which HCT is indicated | Animals  Laboratory samples or cell cultures |
| Interventions | Myeloablative conditioning prior to HCT  Defibrotide  Other interventions aimed at preventing or treating VOD/SOS  Supportive care | Solid organ transplant  Chemotherapy or targeted therapy not used in the context of HCT  Interventions aimed at preventing graft-versus-host disease |
| Outcomes | Accuracy of metrics at predicting or diagnosing VOD/SOS (sensitivity, specificity, positive and negative predictive value, area under the receiver operator curve, and c-statistic)  Recommended assessment procedures for VOD/SOS  Biomarkers, laboratory parameters, and clinical findings associated with VOD/SOS development  Risk factors for VOD/SOS  Reports of VOD/SOS occurrence where metrics used to assess VOD/SOS might be reported  Include any hepatic toxicity as this may be mild VOD/SOS | Non-VOD/SOS outcomes associated with HCT (eg, survival, response rates, and other adverse events) |
| Study methodology | Retrospective or prospective observational studies  Cross-sectional or case-control studies  Database or registry data analyses  Guidelines on the detection and assessment of VOD/SOS  Systematic reviews of relevant studies  Narrative reviews of how to detect and assess VOD/SOS  Full text publications of real-world studies reporting VOD/SOS occurrence | Editorials and letters  Conference abstracts with a corresponding full-text paper and not reporting new data  Clinical trials where detection of VOD/SOS will be based on study protocol and not real-world practice |
| Study size | ≥5 patients with VOD/SOS | <5 patients with VOD/SOS |
| Language | English only |  |
| Publication date | Any |  |

HCT, haematopoietic cell transplantation; VOD/SOS, veno-occlusive disease/sinusoidal obstruction syndrome.

**Appendix Table 4. Additional Embase search to identify guidelines.**

| **Number** | **Search string** | **Hits** |
| --- | --- | --- |
| 1 | 'liver vein obstruction'/exp OR 'liver vein obstruction' OR 'liver venoocclusive disease'/exp OR 'liver venoocclusive disease' OR 'veno-occlusive disease'/exp OR 'veno-occlusive disease' OR 'venoocclusive disease'/exp OR 'venoocclusive disease' OR 'sinusoidal obstruction' OR 'hepatic veno-occlusive disease'/exp OR 'hepatic veno-occlusive disease' | 30,712 |
| 2 | 'stem cell transplantation'/exp OR 'stem cell transplantation' OR 'h?ematopoietic stem cell' OR sct:ab,ti OR hsct:ab,ti OR conditioning:ab,ti OR myeloablati*:ab,ti OR 'bone marrow transplantation'/exp OR 'bone marrow transplantation' OR bmt:ab,ti | 345,598 |
| 3 | 1 AND 2 | 4953 |
| 4 | 1 AND 2 AND [humans]/lim AND [english]/lim AND [abstracts]/lim | 4133 |
| 5 | 'practice guideline'/exp | 619,046 |
| 6 | 4 AND 5 | 143 |

***Search results***

**Appendix Table 5. Diagnostic criteria.**

| **Name** | **Criteria for VOD/SOS diagnosis** | **Reference** |
| --- | --- | --- |
| Seattle | 2 of 3 findings within 30 days of transplantation: bilirubin >34.2 μmol/L; hepatomegaly or RUQ pain of liver origin; ascites with or without unexplained weight gain of >2% over baseline | McDonald GB, et al. Venocclusive disease of the liver after bone marrow transplantation: diagnosis, incidence, and predisposing factors. *Hepatology*. 1984;4(1):116-122. |
| Baltimore | Within 21 days, hyperbilirubinaemia  >34.2 μmol/L plus 2 other criteria: hepatomegaly, usually painful; ≥5% weight gain; and ascites | Jones RJ, et al. Venoocclusive disease of the liver following bone marrow transplantation. *Transplantation*. 1987;44(6):778-783 |
| Modified Seattle | 2 of 3 findings within 20 days of transplantation: bilirubin >34.2 μmol/L; hepatomegaly or RUQ pain of liver origin; and >2% weight gain due to fluid accumulation | Dignan FL, et al. BCSH/BSBMT guideline: diagnosis and management of veno-occlusive disease (sinusoidal obstruction syndrome) following haematopoietic stem cell transplantation. *Br J Haematol*. 2013; 163(4):444-457. |
| EBMT adult criteria | For classic VOD/SOS, use Baltimore criteria; for late-onset VOD/SOS, use the classical VOD/SOS criteria beyond Day 21, or histologically proven VOD/SOS, or ≥2 of the classical criteria and ultrasound or haemodynamical evidence of VOD | Mohty M, et al. Revised diagnosis and severity criteria for sinusoidal obstruction syndrome/veno-occlusive disease in adult patients: a new classification from the European Society for Blood and Marrow Transplantation. *Bone Marrow Transplant*. 2016;51(7):906-912. |
|  |  |  |
| EBMT paediatric criteria | No time limit for onset.  2 or more: unexplained consumptive and transfusion-refractory thrombocytopaenia; otherwise unexplained weight gain on 3 consecutive days despite the use of diuretics, or a weight gain 45% above baseline value; hepatomegaly (best if confirmed by imaging) above baseline value; ascites (best if confirmed by imaging) above baseline value; rising bilirubin from a baseline value on 3 consecutive days or bilirubin ≥2 mg/dL within 72 hours | Corbacioglu S, et al. Diagnosis and severity criteria for sinusoidal obstruction syndrome/veno-occlusive disease in pediatric patients: a new classification from the European Society for Blood and Marrow Transplantation. *Bone Marrow Transplant*. 2018;53(2):138-145. |
| Nanjing criteria | (Abdominal distension and/or RUQ pain) + hepatomegaly + ascites) + confirmed history of pyrrolizidine alkaloid–containing plant use + (elevated bilirubin or abnormal LFTs) + (CT OR MRI evidence of VOD) + evidence excluding competing diagnoses | Zhuge Y, et al; and the Chinese Society of Gastroenterology Committee of Hepatobiliary Disease. Expert consensus on the clinical management of pyrrolizidine alkaloid-induced hepatic sinusoidal obstruction syndrome. *J Gastroenterol Hepatol*. 2019;34(4):634-642. |
| Cairo 2020 | Any 2 of the following criteria following HCT: elevated bilirubin (≥2 mg/L; ≥34.2 μmol/L) or greater than upper institutional limits; unexpected weight gain (≥5% compared to baseline weight pre‐HCT); excessive platelet transfusions consistent with refractory thrombocytopaenia post‐HCT; hepatomegaly for age or increase size over pre‐HCT; RUQ pain; ascites confirmed by physical exam and/or imaging studies; reversal of portal venous flow (hepatofugal flow) by Doppler ultrasound or any 1 of these criteria following HCT plus hepatic biopsy consistent with VOD/SOS or unexplained elevated portal venous wedge pressure | Cairo MS, et al. Modified diagnostic criteria, grading classification and newly elucidated pathophysiology of hepatic SOS/VOD after haematopoietic cell transplantation. *Br J Haematol*. 2020;190(6):822-836. |

VOD/SOS, veno-occlusive disease/sinusoidal obstruction syndrome; RUQ, right upper quadrant; European Society for Blood and Marrow Transplantation (EBMT); LFTs, liver function tests; CT, computed tomography; MRI, magnetic resonance imaging.

**Appendix Table 6. Guidelines publications.**

| **Name** | **Reference** |
| --- | --- |
| Alberta 2021 | Alberta Bone Marrow and Blood Cell Transplant Program: Standard Practice Manual. 15 June 2021. <https://www.albertahealthservices.ca/assets/info/hp/cancer/if-hp-cancer-guide-bmt-manual.pdf>. Accessed 25 March 2022. |
| GITMO 2021 | Bonifazi F, et al. Veno-occlusive disease in HSCT patients: consensus-based recommendations for risk assessment, diagnosis, and management by the GITMO Group. *Transplantation*. 2021;105(4):686-694. |
| NCCN 2021 | National Comprehensive Cancer Network. NCCN guidelines. Acute lymphoblastic leukemia. Version 1.2022. <https://www.nccn.org/professionals/physician_gls/pdf/all.pdf>. |
| Cairo 2020 | Cairo MS, et al. Modified diagnostic criteria, grading classification and newly elucidated pathophysiology of hepatic SOS/VOD after haematopoietic cell transplantation. *Br J Haematol*. 2020;190(6):822-836. |
| EBMT Global Task Force 2020 | Mohty M, et al. Prophylactic, preemptive, and curative treatment for sinusoidal obstruction syndrome/veno-occlusive disease in adult patients: a position statement from an international expert group. *Bone Marrow Transplant*. 2020;55(3):485-495. |
| GITMO 2020 | Botti S, et al. Nursing role in the assessment and care of hepatic sinusoidal obstruction syndrome patients: a consensus paper by the "Gruppo Italiano Trapianto di Midollo Osseo." *Support Care Cancer.* 2020;28(11):5125-5137. |
| NSW 2020 | New South Wales Government. Sinusoidal obstruction syndrome (SOS/VOD) associated with blood and marrow transplantation (BMT). 20 October 2020. <https://www.eviq.org.au/clinical-resources/side-effect-and-toxicity-management/haematological/865-sinusoidal-obstruction-syndrome-sos-vod-asso> |
| PALISI 2020 | Mahadeo KM, et al; and Pediatric Acute Lung Injury and Sepsis Investigators (PALISI) Network and the Pediatric Diseases Working Party of the European Society for Blood and Marrow Transplantation. Diagnosis, grading, and treatment recommendations for children, adolescents, and young adults with sinusoidal obstructive syndrome: an international expert position statement. *Lancet Haematol*. 2020;7(1):e61-e72. |
| Chinese Society of Gastroenterology Committee of Hepatobiliary Disease 2019 | Zhuge Y, et al; and the Chinese Society of Gastroenterology Committee of Hepatobiliary Disease. Expert consensus on the clinical management of pyrrolizidine alkaloid-induced hepatic sinusoidal obstruction syndrome. *J Gastroenterol Hepatol*. 2019;34(4):634-642. |
| EBMT 2019 | Ruutu T, Carreras E. Hepatic complications. In: Carreras E, Dufour C, Mohty M, Kröger N (eds). *The EBMT Handbook: Hematopoietic Stem Cell Transplantation and Cellular Therapies*. 7th ed. Springer; 2019:373-379. |
| EBMT Paediatric 2018 | Corbacioglu S, et al. Diagnosis and severity criteria for sinusoidal obstruction syndrome/veno-occlusive disease in pediatric patients: a new classification from the European Society for Blood and Marrow Transplantation. *Bone Marrow Transplant*. 2018;53(2):138-145. |
| Kebriaei 2018 | Kebriaei P, et al. Management of important adverse events associated with inotuzumab ozogamicin: expert panel review. *Bone Marrow Transplant*. 2018;53(4):449-456. |
| EBMT VOD International Multi-Disciplinary Advisory Board 2017 | Wallhult E, et al. Management of veno-occlusive disease: the multidisciplinary approach to care. *Eur J Haematol*. 2017;98(4):322-329. |
| Middle East/North Africa regional consensus 2017 | Al Jefri AH, et al. Veno-occlusive disease/sinusoidal obstruction syndrome after haematopoietic stem cell transplantation: Middle East/North Africa regional consensus on prevention, diagnosis and management. *Bone Marrow Transplant*. 2017;52(4):588-591. |
| EBMT 2016 | Mohty M, et al. Revised diagnosis and severity criteria for sinusoidal obstruction syndrome/veno-occlusive disease in adult patients: a new classification from the European Society for Blood and Marrow Transplantation. *Bone Marrow Transplant*. 2016;51(7):906-912. |
| National Child Cancer Network New Zealand 2016 | National Child Cancer Network New Zealand. Sinusoidal obstruction syndrome (SOS). 4 May 2016. <https://starship.org.nz/guidelines/sinusoidal-obstruction-syndrome-sos/> |
| EASL 2015 | European Association for the Study of the Liver. EASL Clinical Practice Guidelines: vascular diseases of the liver. *J Hepatol*. 2016;64(1):179-202. |
| EBMT 2015 | Mohty M, et al. Sinusoidal obstruction syndrome/veno-occlusive disease: current situation and perspectives—a position statement from the European Society for Blood and Marrow Transplantation (EBMT). *Bone Marrow Transplant*. 2015; 50(6):781-789. |
| Mahadeo 2014 | Mahadeo KM, Bajwa RPS. Hepatic veno-occlusive disease in children after hematopoietic stem cell transplantation. *J Pediatr Intensive Care*. 2014;3(3):183-193. |
| BCSH/BSBMT 2013 | Dignan FL, et al; and the Haemato-oncology Task Force of British Committee for Standards in Haematology; British Society for Blood and Marrow Transplantation. BCSH/BSBMT guideline: diagnosis and management of veno-occlusive disease (sinusoidal obstruction syndrome) following haematopoietic stem cell transplantation. *Br J Haematol*. 2013;163(4):444-457. |
| AASLD 2009 | DeLeve LD, et al. American Association for the Study Liver Diseases. Vascular disorders of the liver. *Hepatology*. 2009;49(5):1729-1764. |

GITMO, The Italian Group for Bone Marrow Transplantation; HSCT, haematopoietic stem cell transplantation; NCCN, National Comprehensive Cancer Network; VOD/SOS, veno-occlusive disease/sinusoidal obstruction syndrome; EBMT, European Society for Bone and Marrow Transplantation; NSW, New South Wales; PALISI, Paediatric Acute Lung Injury and Sepsis Investigators; EASL, European Association for the Study of the Liver; BCSH, British Committee for Standards in Haematology; BSBMT, British Society of Blood and Marrow Transplantation; AASLD, American Association for the Study of Liver Disease
